# Quantitative MRI Volumetry as a Potential Neuroimaging Biomarker in Levetiracetam-Treated Epilepsy

**DOI:** 10.3390/diagnostics16183013

**Published:** 2026-09-17

**Authors:** Sibel Çıplak, Serkan Ünlü

**Affiliations:** 1 Department of Neurology, Malatya Turgut Özal University Medicine Faculty, Malatya 44210, Turkey; 2 Department of Radiology, Malatya Education and Research Hospital, Malatya 44330, Turkey

**Keywords:** epilepsy, levetiracetam, magnetic resonance imaging, neuroimaging

## Abstract

**Background/Objectives:** Quantitative magnetic resonance imaging (MRI) volumetry may provide an objective approach for detecting subtle longitudinal structural brain changes in patients with epilepsy. This retrospective longitudinal study investigated regional and global brain volume changes observed during levetiracetam (LEV) monotherapy. **Methods**: Thirty-seven adults with epilepsy who underwent brain MRI before initiation of LEV monotherapy (MRI-1) and approximately 12 months later (MRI-2) were included. Automated volumetric segmentation was performed using volBrain, and within-subject changes in macrostructural, subcortical, and cortical volumes were evaluated. **Results:** No significant longitudinal changes were detected in global macrostructural measures, including white matter, gray matter, cerebrospinal fluid, total intracranial cavity, cerebral, cerebellar, brainstem, or ventricular volumes (all *p* > 0.05). In contrast, significant volume reductions were observed in the bilateral putamen, caudate nuclei, and thalami, as well as the left nucleus accumbens, bilateral insular cortex, and left occipital cortex (*p* < 0.05), whereas the right globus pallidus volume increased significantly. **Conclusions:** These findings demonstrate region-specific longitudinal volumetric changes during LEV monotherapy despite preserved global brain volumes. However, because this study lacked a longitudinal healthy control group, the observed changes cannot be definitively attributed to LEV and may also reflect epilepsy-related processes, normal aging, or other clinical factors. Automated MRI volumetry may represent a promising quantitative approach for longitudinal assessment, but its potential biomarker utility requires validation in larger prospective studies incorporating protocol-harmonized healthy controls and detailed clinical and treatment-exposure measures.

## 1. Introduction

Epilepsy is one of the most common neurological diseases, affecting more than 1% of the world’s population, and monotherapy with antiseizure medications (ASMs) forms the basis of treatment, providing seizure control for approximately 70% of patients [1,2]. It is clear that long-term use of ASMs can suppress neuronal excitability in epileptic patients, leading to various problems such as cognitive dysfunction [3]. In recent years, the effects of ASMs on brain morphology, along with seizure control, have received increasing attention. While it is known that morphological changes in cortical and subcortical structures in individuals with epilepsy may be related to the disease, the contribution of the drugs used to these changes has not yet been fully clarified [4,5]. Therefore, evaluating the potential effects of ASMs on brain morphology is important for both understanding the natural course of the disease and correctly interpreting imaging findings.

Magnetic resonance imaging (MRI)-based volumetric analysis methods allow for the quantitative assessment of structural brain changes in neurological diseases. Although manual segmentation has been considered the gold standard method for many years, its use in clinical practice is limited due to its time-consuming nature and observer-dependent variability. In contrast, automated segmentation methods enable more objective and reproducible assessment of global and regional brain volumes [6]. The International League Against Epilepsy (ILAE) emphasizes the importance of quantitative MRI analyses in the assessment of structural brain changes [7].

It has been reported that the effects of ASMs on brain morphology may vary depending on the drug used. Chronic phenytoin use is associated with cerebellar atrophy [8]. Similarly, decreases in total brain volume, white matter volume, and parietal cortical thickness have been reported in patients receiving valproate [9,10]. In an experimental study, transient and reversible changes in brain volume were shown following the administration of valproate and levetiracetam [11]. However, clinical data on the potential effects of levetiracetam on brain volume in humans are limited, and the current findings do not permit a definitive conclusion.

Accordingly, objective and reproducible quantitative imaging approaches may be useful for detecting subtle regional structural changes that are not readily characterized by routine visual MRI assessment. Automated volumetric analysis can provide continuous, region-specific measurements that can be compared longitudinally within the same individual. Such measurements may therefore serve as candidate neuroimaging biomarkers for the detection and monitoring of treatment-associated structural changes. This study aimed to evaluate longitudinal changes in global, subcortical, and cortical brain volumes during levetiracetam monotherapy using automated MRI volumetry and to explore the potential diagnostic and monitoring value of this approach in patients with epilepsy.

## 2. Method

### 2.1. Study Design and Setting

This study was designed as a retrospective, observational, and longitudinal study aiming to compare brain magnetic resonance imaging (MRI) findings obtained before the initiation of levetiracetam (LEV) monotherapy and approximately one year after treatment. The study retrospectively reviewed clinical records and brain MRI images of epilepsy patients followed at the Neurology Outpatient Clinic of Malatya Training and Research Hospital between January 2024 and December 2025. All MRI scans were performed using a 1.5 Tesla Siemens Magnetom Altea device (Siemens Healthineers, Erlangen, Germany). The imaging protocol included thin-section three-dimensional volumetric T1-weighted sequencing along with axial T2-weighted, axial and coronal FLAIR, and TE-zero sequencing.

### 2.2. Patient Selection

The study examined data from 2972 patients aged 18–65 years diagnosed with generalized, focal, onset of unknown, or unclassifiable epilepsy according to the International League Against Epilepsy (ILAE) 2017 classification [12] between 2024 and 2025. 420 patients who underwent two MRI scans at least one year apart were selected. 126 patients using levetiracetam and who underwent two MRI scans at least one year apart were included in the screening.

Patients with lesions indicative of cerebrovascular disease, demyelinating disease, or neurodegenerative disease on brain magnetic resonance imaging; patients diagnosed with intracranial hypertension; patients with a history of intracranial mass; patients with a history of intracranial surgery; patients with a history of head trauma; patients using multiple ASMs; and patients with insufficient magnetic resonance image quality for analysis were excluded from the study, totaling 89 patients.

The inclusion criteria for the study were as follows: age between 18 and 65 years; a diagnosis of generalized, focal, or unknown epilepsy according to the ILAE 2017 classification [12]; using only LEV monotherapy as a single ASM; having at least one brain MRI taken before LEV use at the time of diagnosis; and having a second control MRI taken one year after the start of medication.

**Figure 1:**
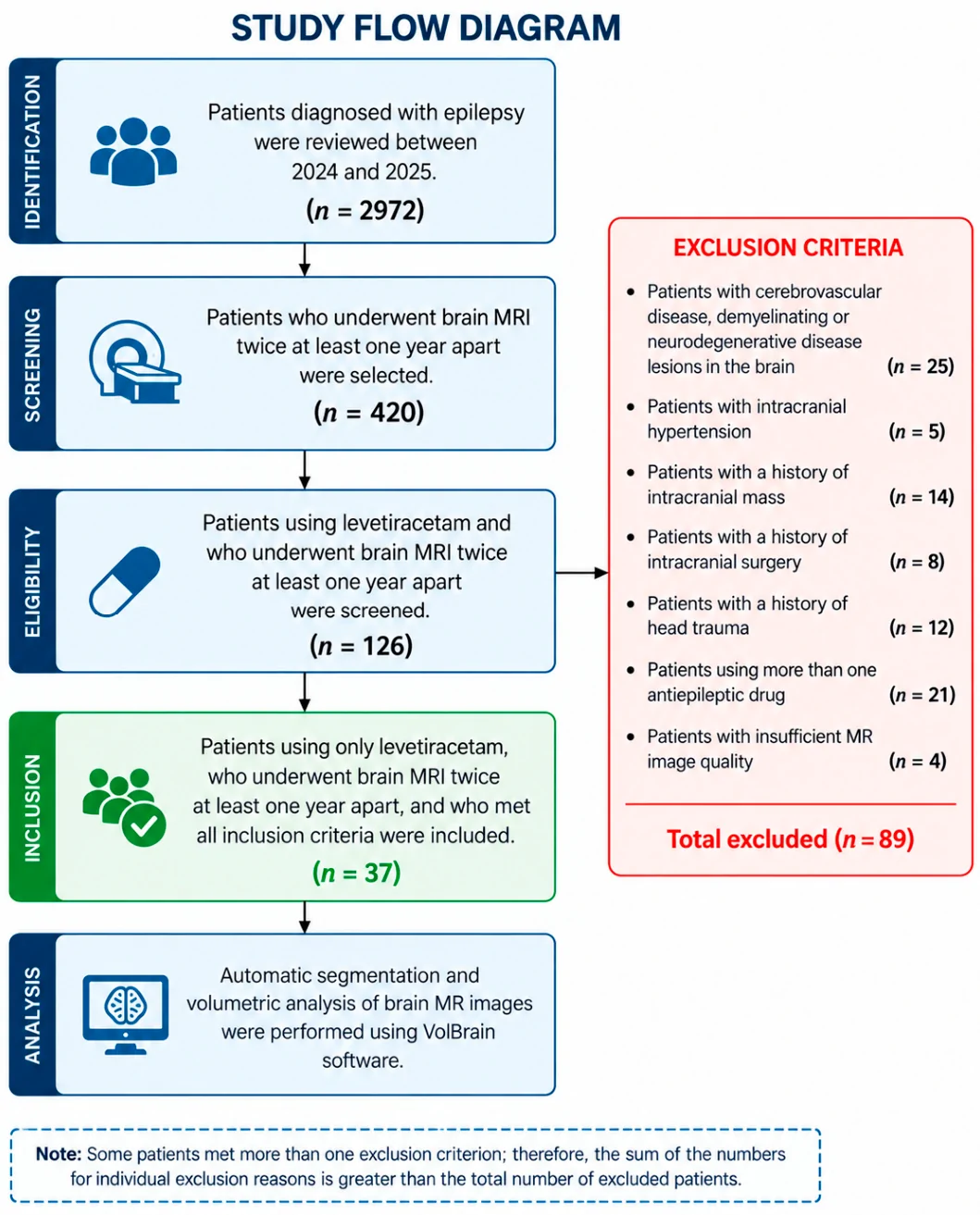
Flow diagram of the patient selection process.

Thirty-seven epilepsy patients meeting the inclusion criteria were included in the study. To reduce selection bias, all consecutive patients meeting the established inclusion criteria were evaluated during the study period (Figure 1).

### 2.3. Sample Size

The sample size of the study was calculated using the G*Power 3.1 program. For paired measurements, a two-way hypothesis, a 5% type I error level (α = 0.05), 80% statistical power (1 − β = 0.80), and an effect size of 0.50 were accepted [13]. Under these assumptions, the minimum sample size required for the dependent samples *t*-test was calculated as 34 patients. A total of 37 patients who met the inclusion criteria were included in the analyses of the present study.

### 2.4. Data Collection

Demographic and clinical data were retrospectively obtained from electronic patient records. The variables evaluated included age, gender, age of seizure onset, duration of epilepsy, seizure type, seizure frequency, medical history, family history, electroencephalography (EEG) findings, duration of LEV use, and MRI dates.

Two separate MRI scans were evaluated for each patient:

MRI-1: baseline image obtained before starting LEV treatment;
MRI-2: control image obtained after approximately 12 months of continuous LEV treatment.

The average time between the two imaging procedures was considered to be approximately 12 months.

There were no missing clinical or imaging data in the patients included in the study.

### 2.5. Volumetric Analysis Method

Volumetric measurements of brain structures were performed using volBrain software (http://volbrain.upv.es/) [6]. VolBrain is a web-based analysis platform with proven accuracy in the literature, which enables automatic segmentation and volumetric analysis of brain MRI images. Measurements are reported as absolute volume values in cubic centimeters (cm^3^) for each structure.

In the analysis, anatomical structures were evaluated in three groups. Macro structures included white matter (WM), gray matter (GM), cerebrospinal fluid (CSF), intracranial cavity (ICC), cerebrum (right/left), cerebellum (right/left), vermis, brainstem, and lateral ventricles (right/left). Subcortical (micro) structures included the putamen (right/left), caudate nucleus (right/left), globus pallidus (right/left), thalamus (right/left), amygdala (right/left), and nucleus accumbens (right/left). Finally, in the cortical lobe analysis, the volumes of the frontal, temporal, parietal, occipital, insular, and limbic cortex were examined separately for each hemisphere.

### 2.6. Biostatistical Data Analyses

Categorical variables are summarized as numbers and percentages. The distribution of quantitative variables was assessed using the Shapiro–Wilk test. Normally distributed variables are expressed as mean ± standard deviation (SD), whereas non-normally distributed variables are summarized as median (minimum–maximum).

A paired-measurement design was used to compare brain volume measurements obtained before initiation of levetiracetam (MRI-1) and after approximately 12 months of continuous LEV monotherapy (MRI-2) in the same patients. Normally distributed paired measurements were compared using the paired-samples *t*-test, whereas non-normally distributed paired measurements were analyzed using the Wilcoxon signed-rank test.

To provide a clinically interpretable measure of longitudinal volumetric change in addition to statistical significance, absolute and percentage changes between MRI-1 and MRI-2 were calculated. Absolute change was defined as MRI-2 minus MRI-1.

Effect sizes were calculated using Cohen’s *d* for paired-samples *t*-tests and the rank-biserial correlation coefficient (*r*) for Wilcoxon signed-rank tests [14]. Cohen’s *d* values of <0.20, 0.20–0.49, 0.50–0.79, and ≥0.80 were interpreted as negligible, small, moderate, and large effects, respectively. Rank-biserial correlation coefficients of <0.10, 0.10–0.29, 0.30–0.49, and ≥0.50 were interpreted as negligible, small, moderate, and large effects, respectively. Paired scatter plots were generated for representative regional and global brain volume measures to visualize individual within-subject volumetric changes between MRI-1 and MRI-2. Individual patient measurements were displayed at both time points and connected by lines to illustrate within-subject trajectories.

Statistical analyses were performed using IBM SPSS Statistics for Windows, version 23.0 (IBM Corp., Armonk, NY, USA) and Python version 3.13.5 (Python Software Foundation, Beaverton, OR, USA), with the SciPy and pandas libraries.

## 3. Results

The mean age of the patients at the initial MRI evaluation was 33.5 ± 14.6 years, and 67.6% were female (*n* = 25). Considering current age, the mean duration of epilepsy was calculated as 6.7 ± 2.3 years. The vast majority of patients had a diagnosis of generalized epilepsy (86.5%). Focal epilepsy was observed in 8.1%, epilepsy of unknown onset in 2.7%, and unclassified epilepsy in 2.7%. Pathological findings were detected on EEG in 35.1% of the patients. The seizure frequency was less than once a month in all patients (Table 1).

In the comparison of MRI-1 and MRI-2, no statistically significant volume change was detected in any macrobrain structure (*p* > 0.05). White matter, gray matter, cerebrospinal fluid, total intracranial cavity volume, and right and left cerebrum, right and left cerebellum, vermis, brainstem, and bilateral lateral ventricle volumes were found to be similar between the two measurements (*p* > 0.05). Effect sizes were small in all macrostructures (Table 2).

### Absolute and Percentage Volume Changes

The tables below report the absolute change (MRI-2 minus MRI-1) and percentage change relative to baseline (MRI-1) for every macrostructural, subcortical, and cortical lobe measure, alongside the *p*-values already reported in the manuscript. For variables analyzed with the paired *t*-test, absolute change is expressed as mean ± SD of within-subject differences, and percentage change is calculated from group means. For variables analyzed with the Wilcoxon signed-rank test, absolute change is expressed as medians (range) of within-subject differences, and percentage change is calculated from group medians, consistent with the descriptive statistics already used elsewhere in the manuscript.

Comparison of the subcortical structures revealed some volume changes (Table 3). Right and left putamen volumes were found to be lower in MRI-2 measurements compared to MRI-1 (right: *p* = 0.0002; left: *p* < 0.0001). Similarly, right and left caudate nucleus volumes (*p* = 0.004 and *p* = 0.037) and bilateral thalamus volumes (*p* = 0.013 for both sides) were lower in MRI-2. A significant decrease was also observed in the volume of the left nucleus accumbens (*p* = 0.030). In contrast, the volume of the right globus pallidus was higher in MRI-2 measurements (*p* = 0.016). There were no statistically significant differences between the two measurements in terms of left globus pallidus, right and left amygdala, and right nucleus accumbens volumes (*p* > 0.05).

When cortical lobe volumes were evaluated, the volumes of the right and left insular cortex were found to be lower in MRI-2 compared to MRI-1 (right: *p* = 0.008; left: *p* = 0.013). A significant decrease was also detected in the volume of the left occipital cortex (*p* = 0.022). The change in the right occipital cortex was not statistically significant (*p* = 0.062). No significant differences were observed between the two measurements in terms of frontal, temporal, parietal, and limbic cortex volumes (*p* > 0.05 for all comparisons) (Table 4).

In addition to the statistical comparisons summarized above, absolute and percentage volume changes were calculated for all regions of interest (Table 2, Table 3 and Table 4). The largest relative reductions were observed in the left putamen (−17.3%), right putamen (−11.2%), bilateral insular cortex (right: −10.9%; left: −11.1%), and left occipital cortex (−7.6%), while bilateral caudate and thalamic volumes decreased by 5.4–8.9%. The left nucleus accumbens decreased by 12.2%. In contrast, the right globus pallidus increased by 7.1%. Global macrostructural measures changed minimally (all |% change| ≤ 7.6%), and none reached statistical significance.

**Figure 2:**
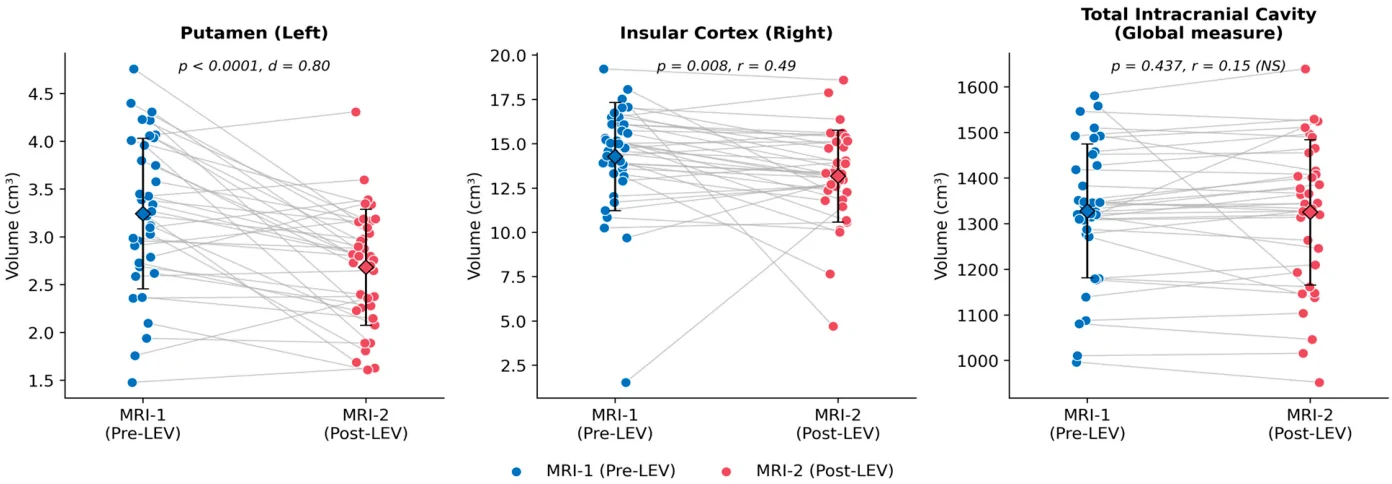
Paired scatter plots of representative regional and global brain volumes before and after levetiracetam monotherapy. Individual patient volumes at MRI-1 (pre-levetiracetam, blue) and MRI-2 (post-levetiracetam, red) for the left putamen, right insular cortex, and total intracranial cavity volume (a global measure). Each dot represents one patient; gray lines connect paired measurements. Diamonds and error bars show group means ± SD.

To further illustrate these regional and global volumetric changes at the individual-subject level, paired scatter plots were generated for two of the most significantly affected structures and for one representative global measure (Figure 2). The left putamen showed a consistent reduction from MRI-1 to MRI-2 (3.24 ± 0.79 cm^3^ vs. 2.68 ± 0.61 cm^3^; paired *t*-test, *p* < 0.0001, Cohen’s *d* = 0.80), with the majority of patients following the same direction of change. Similarly, the right insular cortex volume decreased from a median of 14.76 cm^3^ (range 1.54–19.23) at MRI-1 to 13.15 cm^3^ (range 4.70–18.59) at MRI-2 (Wilcoxon signed-rank test, *p* = 0.008, rank-biserial r = 0.49). In contrast, total intracranial cavity volume, used here as a representative global macrostructural measure, remained essentially unchanged between MRI-1 (median 1332.38 cm^3^, range 996.62–1581.03) and MRI-2 (median 1343.57 cm^3^, range 952.34–1639.69; *p* = 0.437, r = 0.15). Taken together, these individual-level plots visually corroborate the group-level statistics reported in Table 2, Table 3 and Table 4, supporting the interpretation that levetiracetam-associated volumetric changes are regionally specific rather than reflecting a generalized pattern of global brain atrophy.

**Figure 3:**
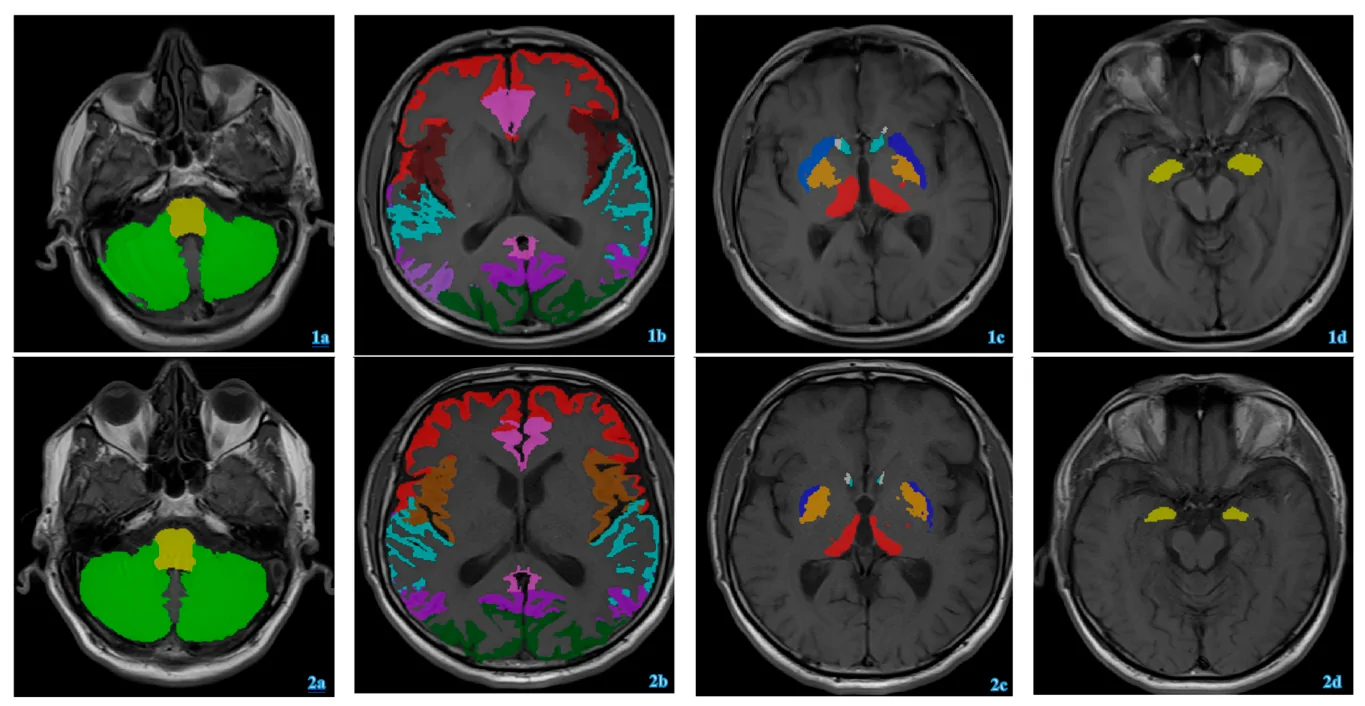
A patient’s MRI-1:a/MRI-2:b: (**1a**,**2a**) cerebellum light green and brainstem yellow; (**1b**,**2b**) frontal red, temporal turquoise, parietal purple, occipital dark green, limbic pink, and insular brown; (**1c**,**2c**) putamen blue, caudate white, pallidum orange, thalamus red, and accumbens turquoise; (**1d**,**2d**) amygdala yellow.

MRI-1 and MRI-2 of a patient examined using the volBrain method are presented below (Figure 3).

## 4. Discussion

In this study, brain MRI images obtained at two different times, approximately one year apart, from 37 epilepsy patients using only levetiracetam were compared using the volBrain method. The majority of patients were diagnosed with generalized epilepsy, and all patients had a seizure frequency of less than once a month. Although no significant volume changes were detected in macrobrain structures, volume reductions were observed in the bilateral putamen, caudate nucleus, thalamus, left nucleus accumbens, bilateral insular cortex, and left occipital cortex. Increases were found in the volume of the right globus pallidus. These results suggest that while overall brain volume is preserved, regional changes may occur in some cortical and subcortical structures.

The absence of significant changes in white matter, gray matter, cerebrospinal fluid, total intracranial volume, cerebral hemispheres, cerebellum, vermis, brainstem, and lateral ventricular volumes suggests that no widespread brain volume loss or ventricular enlargement occurred during the follow-up period. The low seizure frequency in all patients may be a contributing factor to this result. Chambers et al. reported that patients with focal epilepsy with high seizure frequency had lower cortical gray matter, deep gray matter, and white matter volumes; in contrast, patients with low seizure frequency did not differ significantly from healthy controls [15]. All patients had a relatively low and broadly comparable seizure frequency, with less than one seizure per month, which may have reduced the potential influence of differences in seizure burden on longitudinal volumetric measurements. Nevertheless, the present study design does not allow disease-related structural changes to be definitively distinguished from changes potentially associated with levetiracetam treatment. Epilepsy itself, including disease duration, seizure burden, interictal epileptiform activity, and epilepsy subtype, may contribute to progressive cortical and subcortical structural alterations. Therefore, the volumetric differences observed in this study should be interpreted as longitudinal changes occurring during LEV monotherapy rather than as evidence of a causal effect of LEV. The absence of a healthy longitudinal control group further limits our ability to distinguish treatment-associated changes from the natural course of epilepsy or normal age-related structural changes.

The most prominent findings of the present study were the decreases in bilateral putamen, caudate, and thalamus volumes. Large effect sizes were observed, particularly in putamen changes, and moderate-to-large effect sizes were observed in caudate and thalamus changes. Given that most of the patients were diagnosed with generalized epilepsy, these results might be related to the role of thalamocortical and basal ganglia networks in the pathophysiology of epilepsy. Öztürk et al. found that in ESES (electrical status epilepticus in sleep) patients who were not using any ASMs and whose routine MRI examinations were considered normal, bilateral thalamus volumes were lower than both healthy controls and patients with benign childhood centrotemporal spinous epilepsy [16]. This finding suggests that thalamic volume differences might be related to epileptic network activity independently of drug effects.

In the meta-analysis conducted by Nuyts et al., significant volume reductions were reported in the entire brain, putamen, caudate nucleus, pallidum, and supplementary motor area in addition to the thalamus [17]. In this study, partially consistent with these findings, bilateral volume reductions were detected in the putamen, caudate nucleus, and thalamus, while significant volume increases were observed in the right globus pallidus and non-significant volume increases in the right and left amygdala. The volume increase observed in the right globus pallidus indicates that not all components of the basal ganglia exhibited a similar pattern of longitudinal structural change during LEV monotherapy. However, given the observational design of the present study, this finding cannot be attributed specifically to a neuroprotective or volume-increasing effect of levetiracetam.

In a large-sample meta-analysis study by King et al., valproate use was associated with smaller thalamus, hippocampus, and putamen volumes and larger ventricular volume [18]. However, the fact that this study was conducted in patients diagnosed with bipolar disorder and its cross-sectional design limits the direct application of the results to epilepsy patients using levetiracetam. Nevertheless, the study is noteworthy in that it shows that different pharmacological agents can create different morphological patterns in subcortical structures and supports the possibility that the effects of ASMs on structural brain changes may be drug-specific. Importantly, the reductions observed in the bilateral putamen, caudate nuclei, and thalami should not be attributed directly to levetiracetam. Epilepsy itself, particularly disease duration, seizure burden, interictal epileptiform activity, epilepsy subtype, and age-related neuroanatomical changes, may influence regional brain volumes. Previous ASM exposure, residual clinical heterogeneity, and measurement variability associated with automated segmentation may also have contributed to the observed differences. Thus, the present findings demonstrate an association between longitudinal structural changes and the period of LEV monotherapy but do not establish a causal medication effect.

The observed increase in the volume of the right globus pallidus shows a different trend from the decrease in other basal ganglia structures. This finding should be interpreted cautiously due to the lack of significant change in the volume of the left globus pallidus. The increase in the right globus pallidus may be related to compensatory remodeling, anatomical variability, or segmentation differences. Similarly, the small effect-size decrease detected only in the volume of the left nucleus accumbens should be considered a finding requiring confirmation in independent studies. When the cortical lobes were examined, no significant changes were observed in the volumes of the frontal, temporal, parietal, and limbic cortices, while decreases were detected in the bilateral insular cortex and the left occipital cortex. Although the decrease in the right occipital cortex did not reach statistical significance, it showed a moderate effect size. Therefore, it can be considered that the occipital change is not limited to the left hemisphere alone, and the current sample size may be insufficient to show the change on the right side.

In a study by Tondelli et al., cortical thinning and lateral ventricular enlargement were reported in epileptic patients using valproate, particularly in the posterior regions including the cuneus, lingual gyrus, and pericalcarine cortex [19]. The left occipital volume reduction in the present study is partially similar to this finding in terms of region. However, Tondelli et al.’s study evaluated cortical thickness, while our study evaluated lobe volume. In addition, lateral ventricular enlargement was not detected in this study. Therefore, the results of the two studies should not be considered directly equivalent.

Sammarra et al. found no significant cortical or subcortical differences between patients with mild medial temporal lobe epilepsy who received monotherapy and those who did not. However, the treated group showed cortical thinning in some temporal and parietal regions compared to healthy controls. Although no specific conclusions can be drawn about levetiracetam due to the presence of different drugs in the monotherapy group, it has been suggested that some morphological changes observed in epileptic patients may be directly related to epilepsy itself rather than the drug [20]. However, clinical data on the possible effects of levetiracetam on brain structure are still limited. In current studies, it has been reported that levetiracetam treatment in children with centrotemporal spinous childhood epilepsy may be associated with increased cortical thickness in Rolandic areas [21]. In another study, modest improvements were observed in some cognitive areas after levetiracetam treatment in a similar patient group [22]. However, these findings are based on a limited number of pediatric studies and cannot be directly correlated with different epilepsy syndromes. Therefore, the effects of levetiracetam on global or regional brain volume in adult epilepsy patients are not yet sufficiently clear.

In the study by Lv et al. on temporal lobe epilepsy with amygdala enlargement, levetiracetam was given as add-on therapy to patients who did not respond adequately to other drugs, and no significant change in amygdala volume was observed in these patients. However, levetiracetam was not used as monotherapy, and the patients who received the drug formed a more resistant group. Therefore, the study in question does not show the direct morphological effect of levetiracetam [23]. Although the lack of significant change in amygdala volumes in this study is partially consistent with this finding, the patient groups, epilepsy types, and treatment regimens of the two studies are different.

A significant portion of neuroimaging studies examining the effects of ASMs on brain morphology rely on cross-sectional comparisons between treated and untreated patients, or between epilepsy patients and healthy controls [9,21]. Such designs can be affected by variables such as inter-individual anatomical differences, epilepsy type, disease duration, seizure burden, and previous drug exposure. The fact that epilepsy itself is associated with widespread cortical and subcortical changes makes it difficult to determine to what extent the detected differences are disease- or drug-related [4]. Therefore, longitudinal studies in which the same patients are evaluated at different time points are important because they reduce inter-individual variability and allow for direct monitoring of changes over time.

Although the within-subject longitudinal design reduces inter-individual variability related to sex, head size, and baseline anatomy, it does not replace a longitudinal healthy control group. An age- and sex-matched healthy cohort scanned over a comparable time interval would improve the ability to distinguish disease-related, treatment-associated, and normal age-related volumetric changes. We considered the potential use of an external publicly available dataset; however, retrospectively incorporating a cohort acquired using different scanner hardware, field strength, acquisition parameters, image resolution, preprocessing procedures, or segmentation pipelines could introduce systematic site- and scanner-related bias into automated volumetric measurements. For this reason, an external heterogeneous control dataset was not incorporated post hoc. Future prospective studies should therefore include age- and sex-matched healthy controls acquired using the same or prospectively harmonized MRI protocol and analyzed with an identical volumetric pipeline.

The fact that the present study evaluated the same patients at two different times is an important strength in this respect. Using each patient as their own control reduces the effect of gender, head size, and inter-individual anatomical variability. In addition, the use of an automated segmentation method such as volBrain limited observer variability due to manual measurements. Manjón and Coupé reported that volBrain showed high accuracy and reproducibility in segmenting subcortical structures [6]. Mobarak Salari et al. also detected differences in structures such as the hippocampus, thalamus, and cerebellum in epileptic patients compared to healthy controls using volBrain and showed that automated volumetric methods can show subtle structural changes in patients in whom no significant lesions are seen on routine MRI [24].

The potential clinical value of automated quantitative MRI volumetry lies in its ability to transform structural MRI findings from predominantly qualitative observations into objective and reproducible quantitative measurements. In the context of ASM treatment, longitudinal volumetric assessment may provide complementary information for identifying regional structural changes that are not readily characterized by routine visual MRI evaluation. Such quantitative measurements could potentially be incorporated into longitudinal treatment monitoring and, after validation in larger controlled cohorts, may contribute to the development of imaging-based indicators for individualized treatment assessment. In particular, automated measurements of subcortical structures such as the putamen, caudate, and thalamus may represent candidate imaging features for future biomarker development.

This study has several limitations. First, its retrospective, single-center design limits the generalizability of the findings and may have introduced selection bias. Second, although the sample size met the prespecified statistical power requirement, the relatively small cohort may have limited the detection of subtle volumetric changes and does not eliminate the potential influence of residual clinical heterogeneity. Third, the absence of a longitudinal, age- and sex-matched healthy control group prevents definitive differentiation of treatment-associated changes from the natural progression of epilepsy or normal age-related structural changes. Although publicly available datasets such as OpenNeuro may provide healthy MRI data, post hoc incorporation of externally acquired scans with potentially different scanner hardware, acquisition protocols, image resolution, preprocessing procedures, and segmentation conditions could introduce substantial site- and scanner-related bias. Therefore, an external heterogeneous control cohort was not incorporated into the present analysis. Future prospective validation should include protocol-harmonized healthy controls scanned and analyzed under comparable conditions. Furthermore, the approximately one-year follow-up period may not fully reflect long-term structural brain changes. Daily levetiracetam dose, cumulative drug exposure, serum drug levels, and neuropsychological outcomes were not assessed; therefore, the relationship between volumetric changes, treatment exposure, and clinical significance could not be determined. Finally, volumetric measurements were obtained using a single automated segmentation platform without independent validation, which may affect measurement accuracy, particularly in small subcortical structures.

## 5. Conclusions

This longitudinal study demonstrates that automated quantitative MRI volumetry can detect regional structural changes during levetiracetam monotherapy despite preserved global brain volumes. The observed changes in the bilateral putamen, caudate nuclei, thalami, insular cortex, left occipital cortex, and left nucleus accumbens indicate that regional volumetric analysis may provide information beyond conventional global MRI assessment. These findings support the potential role of automated volumetry as a quantitative neuroimaging approach for detecting and longitudinally monitoring regional structural changes observed during ASM treatment. However, the present findings should not be interpreted as demonstrating a causal effect of levetiracetam, and validation in larger prospective studies with protocol-harmonized healthy control groups is required before clinical biomarker utility can be established. In the future, validated imaging biomarkers derived from longitudinal volumetric measurements may contribute to earlier identification of clinically relevant structural changes and individualized treatment monitoring. However, longer-term and larger-scale prospective studies involving more patients are needed.

## Figures and Tables

**Table 1:** Demographic and clinical characteristics.

Characteristics	*n* (%) (*n* = 37)	Mean ± SD/ Median (Min–Max)
Age; MRI-1 (years)	–	33.51 ± 14.58/ 30 (17–63)
Age; MRI-2 (years)	–	34.51 ± 14.58/ 31 (18–64)
Age; Current		40.32 ± 14.41/ 39 (20–67)
Epilepsy duration (years)	–	6.70 ± 2.31/ 7 (2–11)
Gender; Female/Male	25 (67.6%)/12 (32.4%)	–
Epilepsy Type; Generalized/Focal/Unknow	32 (86.5%)/3 (8.1%)/2 (5.4%)	–
EEG Pathology; Absent/Present	24 (64.9%)/13 (35.1%)	–
Seizure Frequency; Less than once a month	37 (100%)	–

SD: standard deviation.

**Table 2:** Macrostructural volumes—absolute and percentage change (MRI-2 vs. MRI-1).

Structure	*n*	Abs. Change (cm^3^)	% Change	*p*	Sig.
White Matter (cm^3^)	37	+10.00 (−209.55 to +526.31)	+1.2%	0.197	NS
Gray Matter (cm^3^)	37	−26.38 (−426.97 to +388.87)	−7.0%	0.101	NS
CSF (cm^3^)	37	+5.90 (−603.06 to +219.38)	−2.1%	0.420	NS
Total ICC (cm^3^)	37	+5.82 (−410.80 to +204.81)	+0.8%	0.437	NS
Cerebrum Right (cm^3^)	37	−1.16 ± 41.23	−0.2%	0.866	NS
Cerebrum Left (cm^3^)	37	−1.21 ± 40.90	−0.2%	0.858	NS
Cerebellum Right (cm^3^)	37	+0.11 (−15.17 to +52.34)	−0.3%	0.769	NS
Cerebellum Left (cm^3^)	37	+0.34 (−16.48 to +51.26)	+0.3%	0.519	NS
Vermis (cm^3^)	37	−0.09 (−3.60 to +5.61)	+1.4%	0.577	NS
Brainstem (cm^3^)	37	+0.04 (−1.89 to +12.94)	+4.8%	0.386	NS
Lat. Ventricle Right (cm^3^)	37	+0.32 (−59.11 to +7.01)	+7.6%	0.331	NS
Lat. Ventricle Left (cm^3^)	37	−0.16 (−50.33 to +6.03)	+2.4%	0.473	NS

Wilcoxon → median (min–max); paired t → mean ± SD; NS: not significant.

**Table 3:** Subcortical volumes—absolute and percentage change (MRI-2 vs. MRI-1).

Structure	*n*	Abs. Change (cm^3^)	% Change	*p*	Sig.
Putamen Right (cm^3^)	37	−0.46 (−2.90 to +2.53)	−11.2%	<0.001	*
Putamen Left (cm^3^)	37	−0.56 ± 0.70	−17.3%	<0.001	*
Caudate Right (cm^3^)	37	−0.20 (−2.92 to +1.48)	−5.4%	0.004	*
Caudate Left (cm^3^)	37	−0.09 (−2.11 to +0.59)	−6.1%	0.037	*
Globus Pallidus Right (cm^3^)	37	+0.12 (−2.18 to +2.65)	+7.1%	0.016	*
Globus Pallidus Left (cm^3^)	37	+0.13 (−1.18 to +3.04)	+4.9%	0.124	NS
Thalamus Right (cm^3^)	37	−0.44 (−6.28 to +2.55)	−8.9%	0.013	*
Thalamus Left (cm^3^)	37	−0.43 (−7.00 to +2.51)	−6.5%	0.013	*
Amygdala Right (cm^3^)	37	+0.11 (−1.54 to +0.93)	+4.9%	0.057	NS
Amygdala Left (cm^3^)	37	+0.10 (−1.79 to +1.14)	+2.0%	0.319	NS
N. Accumbens Right (cm^3^)	37	−0.02 ± 0.11	−8.8%	0.228	NS
N. Accumbens Left (cm^3^)	37	−0.03 ± 0.09	−12.2%	0.030	*

Wilcoxon test: median (min–max); paired *t*-test: mean ± SD. * *p* < 0.05; NS: not significant.

**Table 4:** Comparison of the cortical lobe volumes using MRI-1 and MRI-2.

Structure	*n*	Abs. Change (cm^3^)	% Change	*p*	Sig.
Frontal Right (cm^3^)	37	−2.47 (−50.77 to +67.62)	−8.4%	0.213	NS
Frontal Left (cm^3^)	37	−1.53 (−50.53 to +64.88)	−6.6%	0.362	NS
Temporal Right (cm^3^)	37	−1.39 (−47.65 to +29.38)	−3.0%	0.239	NS
Temporal Left (cm^3^)	37	−1.42 (−42.65 to +24.33)	−1.9%	0.473	NS
Parietal Right (cm^3^)	37	−1.24 (−35.99 to +38.90)	−4.0%	0.106	NS
Parietal Left (cm^3^)	37	−2.44 (−31.24 to +37.91)	−4.5%	0.169	NS
Occipital Right (cm^3^)	37	−1.08 (−23.99 to +6.49)	−4.9%	0.062	NS
Occipital Left (cm^3^)	37	−2.31 (−23.64 to +7.66)	−7.6%	0.022	*
Insular Cortex Right (cm^3^)	37	−0.65 (−12.83 to +9.91)	−10.9%	0.008	*
Insular Cortex Left (cm^3^)	37	−1.03 (−14.02 to +9.84)	−11.1%	0.013	*
Limbic Cortex Right (cm^3^)	37	−0.45 (−17.34 to +13.57)	−4.8%	0.502	NS
Limbic Cortex Left (cm^3^)	37	−0.56 (−16.86 to +14.27)	−5.3%	0.464	NS

Wilcoxon signed-rank test; median (min–max). * *p* < 0.05; NS: not significant.

## Data Availability

Data supporting the findings of this study are available from the corresponding author upon reasonable request.
